# HIF‐1α‐induced up‐regulation of microRNA‐126 contributes to the effectiveness of exercise training on myocardial angiogenesis in myocardial infarction rats

**DOI:** 10.1111/jcmm.15892

**Published:** 2020-09-16

**Authors:** Wei Song, Qiaoqin Liang, Mengxin Cai, Zhenjun Tian

**Affiliations:** ^1^ Institute of Sports and Exercise Biology Shaanxi Normal University Xi'an China

**Keywords:** exercise training, HIF‐1α, miR‐126, myocardial angiogenesis, myocardial infarction

## Abstract

Exercise training (ET) is a non‐drug natural rehabilitation approach for myocardial infarction (MI). Among the numerous beneficial effects of ET, myocardial angiogenesis is indispensable. In the present study, we investigated the role and mechanism of HIF‐1α and miR‐126 in ET‐induced MI myocardial angiogenesis which may provide new insights for MI treatment. Rat model of post‐MI and human umbilical vein endothelial cells (HUVECs) were employed for our research. Histomorphology, immunohistochemistry, quantitative real‐time PCR, Western blotting and small‐interfering RNA (siRNA) transfection were applied to evaluate the morphological, functional and molecular mechanisms. In vivo results showed that 4‐week ET could significantly increase the expression of HIF‐1α and miR‐126 and reduce the expression of PIK3R2 and SPRED1, while 2ME2 (HIF‐1α inhibitor) partially attenuated the effect of ET treatment. In vitro results showed that HIF‐1α could trigger expression of miR‐126 in HUVECs in both normoxia and hypoxia, and miR‐126 may be involved in the tube formation of HUVECs under hypoxia through the PI3K/AKT/eNOS and MAPK signalling pathway. In conclusion, we revealed that HIF‐1α, whose expression experiences up‐regulation during ET, could function as an upstream regulator to miR‐126, resulting in angiogenesis promotion through the PI3K/AKT/eNOS and MAPK signalling pathway and subsequent improvement of the MI heart function.

## 1 INTRODUCTION

According to *Report on Cardiovascular Diseases in China* (2018), 290 million patients are suffering from cardiovascular diseases, which ranks first by accounting for 44% in overall disease death in China.^1^ Myocardial infarction (MI), a severe ischaemic heart disease, refers to myocardial necrosis caused by coronary hypoxic ischaemia and is a primary cause of death among all types of cardiovascular diseases.^2^ Currently, traditional therapies, including medication, interventional and surgical therapy, play an irreplaceable role in MI treatment. However, as the last link of post‐MI treatment, cardiac rehabilitation is of great significance to improve patients' life quality and reduce mortality rates. Among all the research previously carried out, exercise training (ET) constitutes an important part and is consistently identified as a central element in cardiac rehabilitation.^3^ Increasing evidence indicates the protective effect of ET against diverse cardiovascular diseases, and ET is thus considered an acceptable method for the active role in the rehabilitation of MI hearts.^4^ Exercise, a non‐drug natural cardiac rehabilitation means,^5^ can boast of its superiorities for its simple administration, easy application and lack of obvious side effects. Yet up to now, the mechanism of ET‐induced MI cardiac protection has not been fully elucidated.

Myocardial angiogenesis has been recognized as one of the innovative therapeutic methods for the treatment of MI. Angiogenesis refers to the generation of new blood vessels from pre‐existing capillaries,^6^ which is regulated by a wide range of regulators and signalling molecules.^7^ Among the molecules involved in the angiogenesis induced by ET, hypoxia‐inducible factor‐1 α (HIF‐1α) may play a crucial role.^8^ HIF‐1α is an important transcription factor that regulates angiogenesis through transcription of a series of hypoxia response genes archived with hypoxia response element.^9^ Acute exercise is accompanied by reduced regional‐and‐systemic partial pressure of oxygen, which are stimulatory factors of HIF‐1α.^10^ Ten‐week aerobic exercise has been proven to be able to significantly up‐regulate the expression of HIF‐1α in rat left ventricle.^11^ Therefore, we speculated that HIF‐1α would play important role in ET‐induced myocardial angiogenesis and cardiac function improvement in MI heart.

MicroRNAs (miRNAs; miRs) are small (18‐25 nucleotides in length), single‐stranded non‐coding RNAs that act as post‐transcriptional regulators of gene expression. It has been discovered that dozens of miRNAs, such as miR‐221/222, miR‐17‐72 cluster, miR‐126 and miR‐93, are involved in various stages of angiogenesis.^12^, ^13^, ^14^ Among them, miR‐126, highly expressed in vascular endothelial cells, is considered a master regulator of physiological angiogenesis and vascular integrity.^15^ miR‐126 functions by directly repressing two negative regulators, phosphoinositol‐3 kinase regulatory subunit 2 (PIK3R2) and Sprouty‐related protein 1 (SPRED1).^16^ PIK3R2 and SPRED1 have been shown to function as angiogenesis signalling via PIK3/Akt/eNOS and Ras/MAPK pathway, respectively.^17^ Loss of miR‐126 causes leaky vessels and haemorrhage in zebrafish during embryonic development.^18^ Furthermore, targeted deletion of miR‐126 in mice causes partial embryonic or perinatal lethality and the proliferation of ECs in embryos is significantly reduced. Our previous research found that ET could significantly up‐regulate the expression of miR‐126 of MI myocardium, suppress its target protein expression of PIK3R2/SPRED1 and promote angiogenesis in peri‐infarct zone,^19^ leaving the mechanism that ET‐induced up‐regulation of miR‐126 unexplored fully. As it is well established that some miRNAs are identified to be regulated under hypoxic conditions,^20^ we then hypothesize that ET may contribute to myocardial angiogenesis against MI injury via the HIF‐1α/miR‐126 pathway.

In this study, rat model of MI and human umbilical vein endothelial cells (HUVECs) were employed to valid our hypothesis, and we explored the role of HIF‐1α and miR‐126 in ET‐induced cardiac protection of MI hearts and its underlying mechanisms. Hopefully, these findings can provide new targets for the research on the rehabilitation mechanism of ischaemic heart diseases.

## 2 MATERIALS AND METHODS

### 2.1 Animal

A total of 50 male Sprague Dawley rats (204 ± 6 g, SPF) were purchased in the Laboratory Animal Centre of Xi'an Jiao tong University (Animal certificate No.: SCXK2012‐201). Rats were housed five per cage in a temperature‐controlled room with a 12‐hour dark‐light cycle and free access to water and standard rat chow. All experimental protocols were approved by the Review Committee for the Use of Human or Animal Subjects of Shaanxi Normal University.

### 2.2 Surgical procedures and experimental design

Experimental MI models were induced by the ligation of the left anterior descending coronary artery (LAD) as previously research applied.^21^ Briefly, the rats were anaesthetized by intraperitoneal (ip) injection of sodium pentobarbital (30 mg/kg body weight). LAD was ligated under the stereoscopic microscope (OLYMPUS SZX16), 2 mm beneath the left atrial appendage. Sham‐operated rats had the same surgery, with threading yet no ligation. Four rats died during or after the operation, and two rats without ST elevation were eliminated. All post‐operation rats were randomly assigned to five groups: sham‐operated group (S, n = 8), myocardial infarction group (MI, n = 9), MI‐with‐ET group (MIE, n = 9), MIE group treated with HIF‐1α inhibitor 2‐methoxyestradiol (2ME2; MIE + 2ME2, n = 9) and MIE group treated with PBS (MIE + PBS, n = 9).

### 2.3 Drug administration

The specific HIF‐1α inhibitor 2‐methoxyestradiol (2ME2; Selleck Chemicals) was dissolved in PBS with 10% dimethy1 sulfoxide (DMSO). 2ME2 was administered at 15 mg/kg by intraperitoneal injection 10 minutes before ET in MIE + 2ME2 group.^22^ Rats in MIE + PBS group received the same volume of vehicle (10% DMSO in PBS). All rats in the two groups were weighed once a week to determine the next week dose.

### 2.4 Exercise training protocols

**Figure 1:**
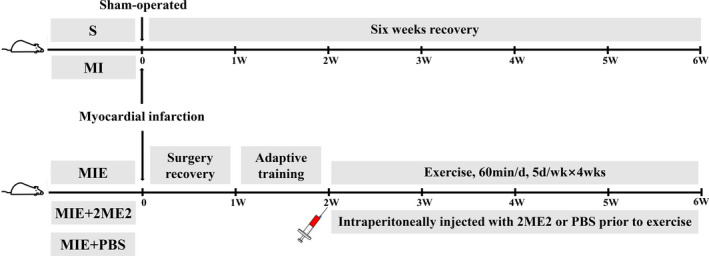
Experimental animal programmes. After the surgery, rats in S and MI groups will receive rest‐treatment until the end of the whole experiment. Rats in MIE, MIE + 2ME2 and MIE + PBS groups will experience one‐week surgery recovery and another week of adjustment exercise, followed by formal ET programme for next four weeks. S, sham‐operated group; MI, myocardial infarction group; MIE, MI‐with‐ET group; MIE + 2ME2, MIE group treated with HIF‐1α inhibitor 2‐methoxyestradiol (2ME2); MIE + PBS, MIE group treated with PBS

Seven days after the surgery, MIE, MIE + 2ME2 and MIE + PBS rats underwent one week of adaptive training (15 m/min, 30 min/d × 5 d) on a motorized rodent treadmill (ZH‐PT, Zheng Hua, An Hui, China), and then all rats ran for 3 minutes at 15 m/min (50% ~ 60% VO_2 max_) followed by 4 minutes at 25 m/min (85% ~ 90% VO_2 max_), which was repeated seven times, 60 min/d (including warm‐up and cooling down), 5 d/wk × 4 wk. Two rats were removed because they could not adapt to the running pace. Three rats died during the ET, possibly as a result of severe heart failure. According to the preparation of MI model and their post‐exercise intervention survival, the number of rats in each group was 7 (Figure 1).

### 2.5 Hemodynamic measurement

The next day after the 4 weeks' training, all rats were anaesthetized as mentioned above, and then, the right carotid artery was isolated and cannulated with a pressure catheter. The tip of the catheter was advanced from the aorta to the left ventricular (LV) cavity. LV cardiac function was obtained via intraventricular catheter recordings (Powerlab 8/30™, ML 870; AD Instruments). The following parameters were measured: LV end‐diastolic pressure (LVEDP), LV systolic pressure (LVSP), maximal positive and minimal negative first derivative of LV pressure (±dp/dtmax).

### 2.6 Histomorphology analysis of collagen deposition

After the haemodynamic measurement, thoracotomy was conducted immediately and hearts were collected. For histological staining, heart samples were fixed in ice‐cold 4% paraformaldehyde for 48 hours, routinely paraffin embedded sectioned (5 μm). Masson's trichrome staining was applied to evaluate myocardium collagen deposition, and collagen volume fraction (CVF) was measured. CVF was quantified by calculating the area percentage of collagen staining performed with Image‐Pro plus 6.0 software. CVF = collagen area/total area. Five visions under a microscope of each sample were randomly chosen, and the average of them was taken for analysis.

### 2.7 Immunofluorescence labelling

After antigen retrieval, the paraffin sections were incubated with the following diluted antibodies overnight at 4°C: mouse monoclonal anti‐proliferating cell nuclear antigen antibody (PCNA; 1:50 dilution; CST), anti‐Von Willebrand factor polyclonal antibody (vWF; 1:400 dilution; Abcam). After being washed with PBS (three times, 5 minutes each time), FITC/TRITC‐conjugated goat anti‐rabbit/mouse IgG antibody (1:100 dilution; Jackson Immuno Research) and 4′6‐diamidino‐2‐phenylindole (DAPI; 1:800; Sigma) were incubated with the tissue sections at 37°C for 1 hour. The images of immunofluorescence staining were obtained by a fluorescence microscope (Nikon Eclipse 55i; Nikon).

### 2.8 Cell culture and hypoxia treatment

Human umbilical vein endothelial cells (HUVECs) were purchased from American Type Culture Collection (ATCC). The cells were maintained in DMEM (Gibco‐BRL), supplemented with 10% newborn calf serum (Invitrogen Life Technologies) and 1% penicillin‐streptomycin (Invitrogen; Thermo Fisher Scientific) at 37°C in a humidified atmosphere with 5% CO_2_ and 21% O_2_ (371; Thermo Fisher Scientific). The cells were treated with different concentrations of HIF‐1 signalling pathway activator dimethyloxalylglycine (DMOG, 10, 40 and 70 μmol; Selleck Chemicals) and 2ME2 (50, 100 and 200 μmol), respectively, for 6 hours before hypoxia treatment.^23^ To induce hypoxia, HUVECs were cultured in the atmosphere of 1% O_2_, 5% CO_2_ and 94% N_2_ (3131; Thermo Fisher Scientific) for 2 hours.

### 2.9 Tube formation assay

All required groups of HUVECs were prepared for culture. 24‐well plate, pipette tips and matrix gels were pre‐cooled the day before the test. 300 μL Matrigel matrix (BD Biosciences) was added to 24‐well plate and put into incubator for gel formation. Subsequent to gel solidification (30 minutes), 500 μL of HUVECs suspension (2 × 10^5^/mL) was added to each well. The plate was incubated at 37°C in the corresponding incubator. After incubation for 4 hours, the branch points of the formed tubes were observed and quantitated at the 100 × magnification using an inverted microscope (DMIL LED; Leica). ImageJ software was used to analyse the tube lengths in 5 randomly selected visual fields.

### 2.10 Transfection

Has‐miR‐126 mimics and has‐miR‐126 inhibitors were ordered from GenePharma. Their sequences were as follows: has‐miR‐126 mimics sense 5′‐UCGUACCGUGAGUAAUAAUGCG‐3′ and antisense 5′‐CAUUAUUACUCACGGUACGAUU‐3′; negative control sense 5′‐UUCUCCGAACGUGUCACGUTT‐3′ and antisense 5′‐ACGUGACACGUUCGGAGAATT‐3′; has‐miR‐126 inhibitors: 5′‐CGCAUUAUUACUCACGGUACGGA‐3′; mircroRNA inhibitor N.C 5′‐CAGUACUUUUGUGUAGUACAA‐3′; negative control FAM sense 5′‐UUCUCCGAACGUGUCACGUTT‐3′ and antisense 5′‐ACGUGACACGUUCGGAGAATT‐3′; mircroRNA inhibitor N.C‐FAM 5′‐CAGUACUUUUGUGUAGUACAA‐3′. They were transfected into HUVECs by Lipofectamine 2000 (Invitrogen Life Technologies) according to the manufacturer's instructions. The transfection efficiency was determined by fluorescently labelled negative control FAM and mircroRNA inhibitor N.C‐FAM (DMIL LED; Leica).

### 2.11 Western Blotting

The frozen hearts and HUVECs were lysed using RIPA lysis buffer, and then, protein concentration was determined using BCA kit. Protein samples were separated by 8%‐10% SDS‐PAGE gels (Bio‐Rad), transferred to a nitrocellulose membrane (Millipore) and immunoblotted with HIF‐1α antibody (1:800; Abcam), PIK3R2 (1:500, Signalway Antibody), SPRED1 (1:800, Merck Millipore), PI3K (1:1500; Cell Signaling), p‐PI3K (1:800; Cell Signaling), AKT (1:1000; Cell Signaling), p‐AKT (1:2000; Cell Signaling), eNOS(1:800; Abcam), p‐eNOS (1:1000; Abcam), Raf‐1 (1:1200; Abcam), ERK (1:1000; Cell Signaling) and p‐ERK (1:1500; Cell Signaling). GAPDH (1:10 000; Bioworld) was used as loading control to determine the relative expression level of the target protein. A full‐feature instrument (Bio‐Rad Chemidoc MP) was used for gels imaging and analysing.

### 2.12 Quantitative real‐time polymerase chain reaction

Total RNA from cardiac tissues and HUVECs were extracted using TRIzol reagent (Life Technologies). cDNA products were obtained from the real‐time fluorescence quantitative PCR instrument (Bio‐Rad CFX96), in accordance with the operational steps of the reverse transcription Kit (miScript II RT Kit; Qiagen). The level of miR‐126 was tested using miScript SYBR Green PCR Kit (Qiagen). U6 was used as an endogenous control. Forward and reverse primers of miR‐126 and U6 were provided by Qiagen. Quantification of relative gene expression was calculated by the comparative Ct method (2^−△△^ *^C^* ^t^) as described by the manufacturer.

### 2.13 Statistical analysis

Data were expressed as mean ± standard deviation. The SPSS 20.0 was used for general statistical analysis. The differences between groups were performed with one‐way ANOVA followed by Tukey's post hoc test. A two‐sided *P* value less than .05 was accepted as statistically significant. Masson stained sections were observed and photographed under light microscope (BX51 OLYMPUS), and CVF was measured by Image‐Pro plus 6.0 software. Histograms were plotted by GraphPad Prism 5.01 software.

## 3 RESULTS

### 3.1 Effect of ET and ET combined 2ME2 on cardiac function and MI‐induced ventricular pathological remodelling

**Figure 2:**
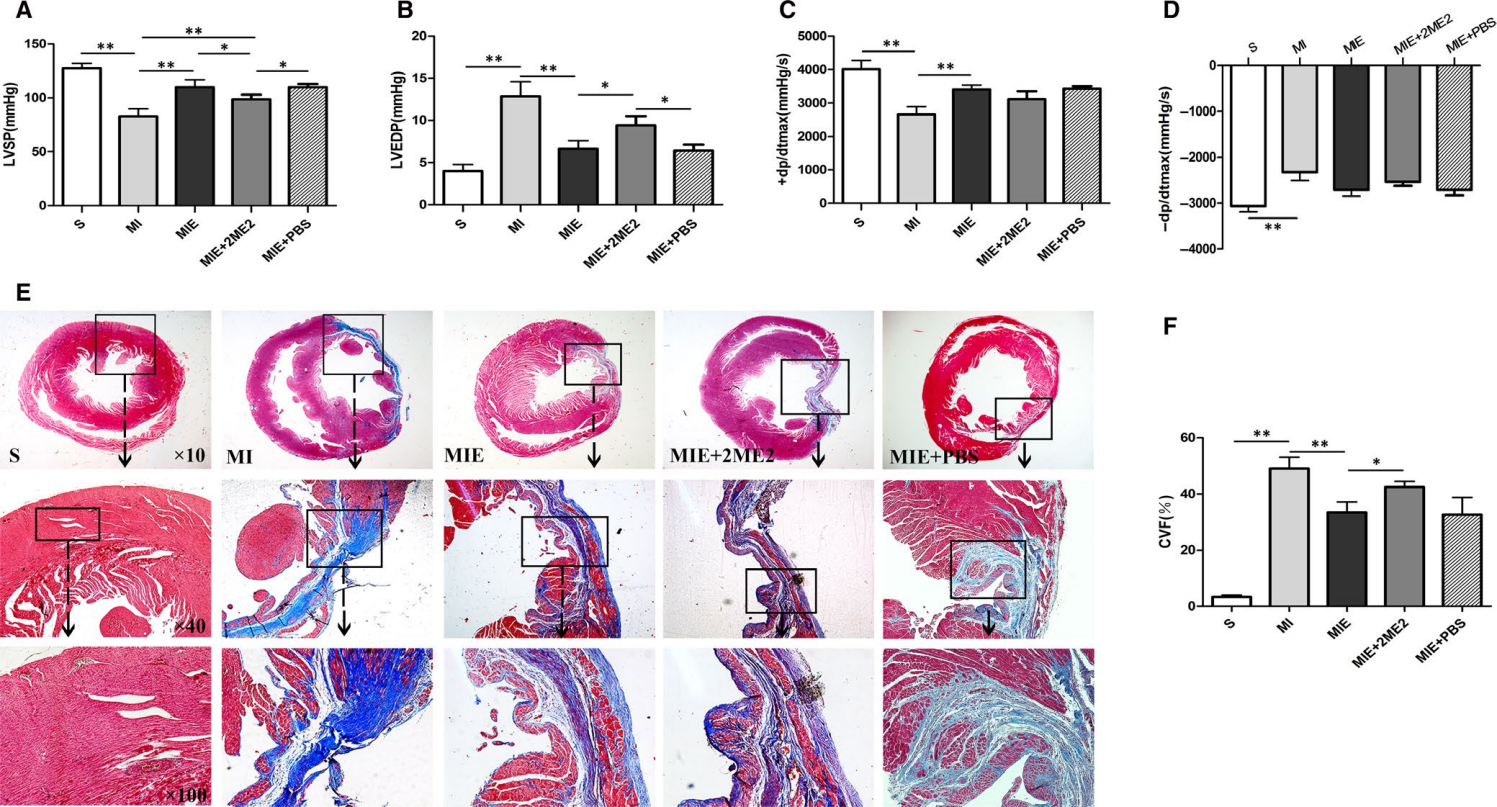
Cardiac function and myocardial fibrosis of rats in S, MI, MIE, MIE + 2ME2, MIE + PBS groups. (A‐D) Haemodynamic measurement of cardiac function (n = 7/group). LVSP, left ventricular systolic pressure; LVEDP, left ventricular end‐diastolic pressure; +dp/dtmax, maximal rate of left ventricular pressure rise; −dp/dtmax, maximal rate of left ventricular pressure fall. (E) Masson's trichrome staining of cardiac tissue (×10, ×40, ×100). Collagen is stained blue, indicating fibrosis. The area of collagen deposition in the peri‐infarcted zones of heart is evaluated by the collagen volume fraction (CVF). (F) The graph showing a statistical analysis of CVF. Values are expressed as mean ± SD (n = 3/group), *P < .05; **P < .01

To evaluate the effect of ET and ET combined 2ME2 on cardiac hemodynamics, ventricular function was performed in all groups. There was a significant reduction in LVSP, +dp/dt_max_ and −dp/dt_max_ in MI group compared with S group (*P* < .01, Figure 2A,C,D); however, LVEDP was significantly increased in the MI group (*P* < .01). 4‐week ET significantly increased LVSP, +dp/dt_max_ (*P* < .01) and decreased LVEDP (*P* < .01) compared with MI group (Figure 2A‐C). ET combined 2ME2 significantly decreased LVSP (*P* < .05) and increased LVEDP (*P* < .05) compared with MIE group (Figure 2A‐B). To investigate ET and ET combined 2ME2 on cardiac remodelling, we determined the myocardial fibrotic response in all groups. As shown in Figure 2E, the collagen fibres were blue, the cardiomyocytes red and the nucleuses blue violet. CVF was significantly increased in MI group compared with S group (*P* < .01). 4‐week ET significantly reduced CVF compared with MI group (*P* < .01). CVF in MIE + 2ME2 group was significantly higher than that in MIE group (*P* < .05, Figure 2F). Together, these data have shown that cardiac function was impaired after MI; ET could improve the cardiac function of MI heart, while 2ME2 partially attenuated the cardiac protective effect of ET treatment.

### 3.2 Effect of ET and ET combined 2ME2 on myocardial angiogenesis

**Figure 3:**
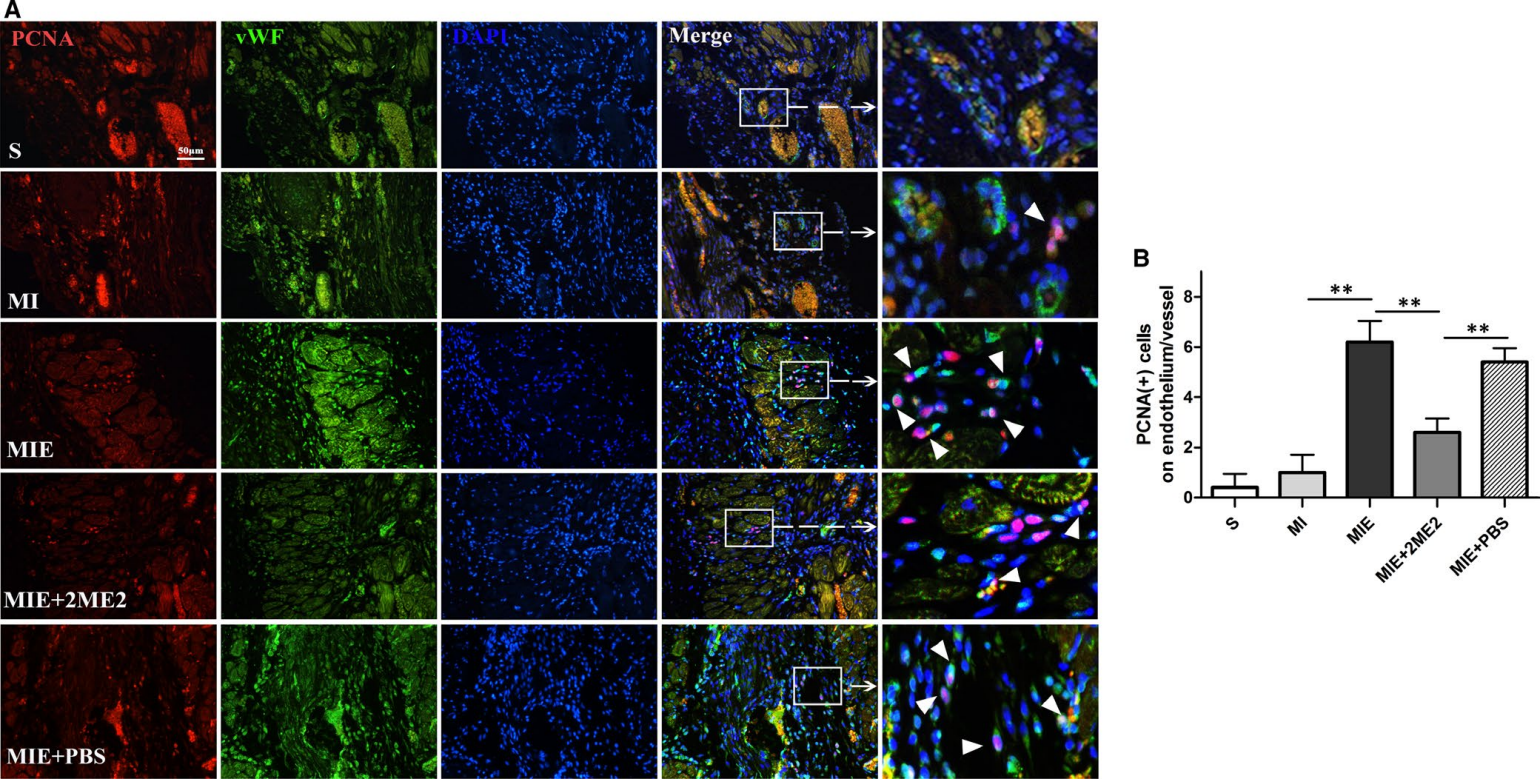
Myocardial angiogenesis of rats in S, MI, MIE, MIE + 2ME2, MIE + PBS groups. (A) Immunofluorescence co‐staining of PCNA (red fluorescence), vWF (green fluorescence) and DAPI (blue fluorescence). Scale bar =  50  μm.(B) The graph showing a statistical analysis of the number of PCNA^+^/vWF^+^ cells. Values are expressed as mean ± SD (n = 3/group), *P < .05; **P < .01

To evaluate the effect on myocardial angiogenesis, we analysed endothelial cell proliferation by co‐staining of PCNA^+^ and vWF^+^, a key factor in DNA replication and a glycoprotein produced by endothelial cells respectively. In S group tissue, almost no PCNA^+^/vWF^+^ cells were detected. But strong staining was detected in other four groups (Figure 3A). Compared with MI group, 4‐week ET induced more PCNA^+^/ vWF^+^ cells (*P* < .01). Whereas, the double stained cells were significantly reduced in MIE + 2ME2 group compared with MIE group (*P* < .01, Figure 3B). All the above results indicated that 4‐week ET could promote myocardial angiogenesis in post‐MI heart, while 2ME2 partially attenuated the protective effect of ET treatment.

### 3.3 Effect of IE and IE combined 2ME2 on expression of HIF‐1α, miR‐126 and its targets

**Figure 4:**
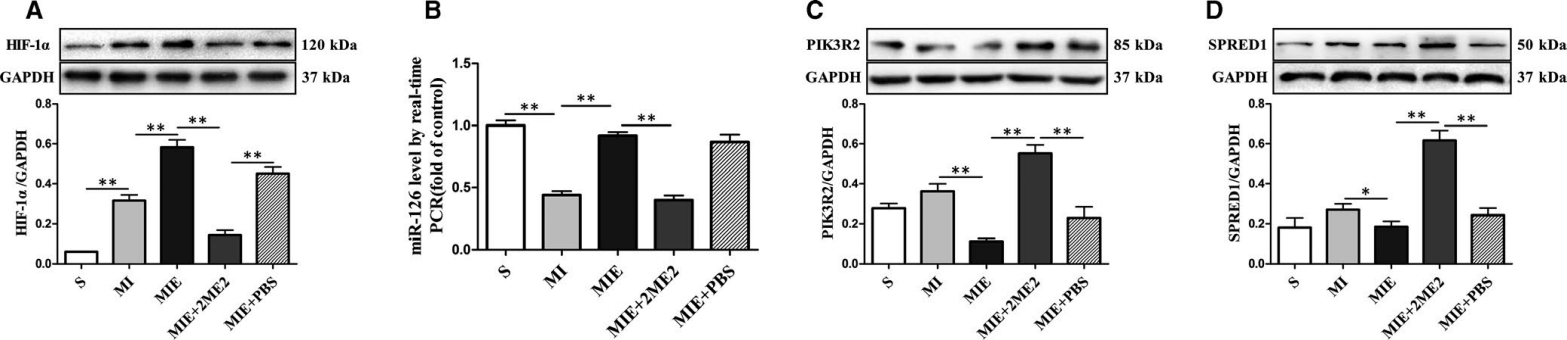
Expression of HIF‐1α, miR‐126 and its targets of rats in S, MI, MIE, MIE + 2ME2, MIE + PBS groups. (A) Protein expressions of HIF‐1α in the peri‐infarcted zones of heart were measured by Western blot. (B) Expression of miR‐126 in the peri‐infarcted zones of heart were measured by RT‐qPCR. (C) Protein expressions of PIK3R2 in the peri‐infarcted zones of heart were measured by Western blot. (D) Protein expressions of SPRED1 in the peri‐infarcted zones of heart were measured by Western blot. Values are expressed as mean ± SD (n = 4/group), *P < .05; **P < .01

The protein expressions of cardiac HIF‐1α were detected using Western blot for all groups. Compared with MI group, the expression of HIF‐1α was significantly up‐regulated in MIE group (*P* < .01). Additionally, HIF‐1α underwent a significant down‐regulation (*P* < .01) in MIE + 2ME2 group compared with MIE group (Figure 4A). Cardiac miR‐126 expression was analysed by real‐time polymerase chain reaction (RT‐qPCR) for all groups. Figure 4B showed increased miR‐126 expression (*P* < .01) in MIE groups compared with MI group. miR‐126 was significantly down‐regulated (*P* < .01) in MIE + 2ME2 group compared with MIE group. As we have predicted, the change tendency in PIK3R2 and SPRED1 protein levels were inversely proportional to miR‐126 expression (Figure 4C‐D). All the above results indicated that ET could increase the expression of HIF‐1α and miR‐126 and reduce the expression of PIK3R2 and SPRED1, which could be all attenuated by 2ME2.

### 3.4 Effect of DMOG and 2ME2 on expression of HIF‐1α, miR‐126 and its targets in HUVECs

**Figure 5:**
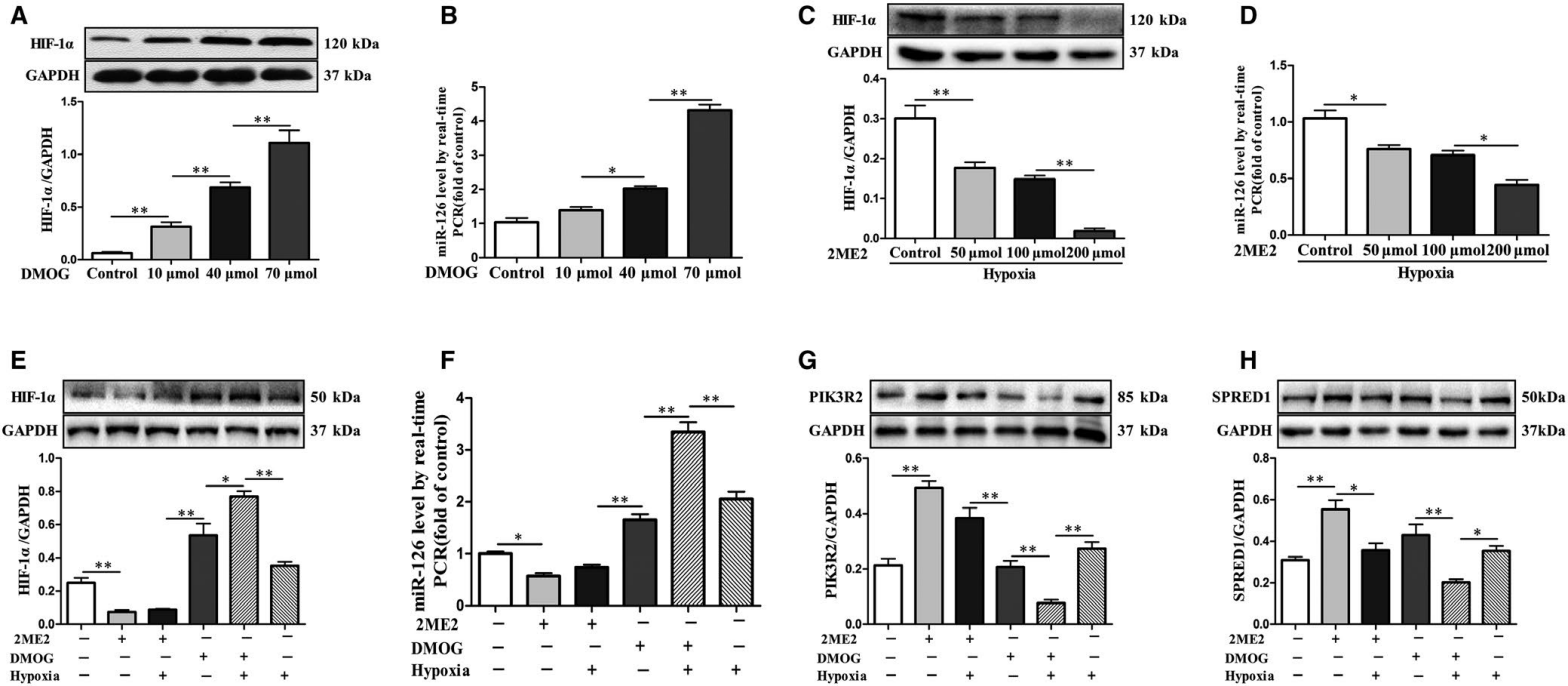
Expression of HIF‐1α, miR‐126 and its targets in HUVECs treated with DMOG or 2ME2. The expressions level of HIF‐1α (A) and miR‐126 (B) in HUVECs treated with different concentrations of DMOG (10, 40 and 70 μmol) for 6 hours in normoxia. The expressions level of HIF‐1α (C) and miR‐126 (D) in HUVECs treated with different concentrations of 2ME2 (50, 100 and 200 μmol) for 6 hours, followed by 2 hours in hypoxia. The expressions level of HIF‐1α (E), miR‐126 (F), PIK3R2 (G) and SPRED1 (H) in the HUVECs treated with 2ME2 (200 μmol) or DMOG (70 μmol) in either normoxia or hypoxia. Values are expressed as mean ± SD (n = 3/group), *P < .05; **P < .01

To explore whether HIF‐1α might influenced miR‐126 expression, HUVECs were treated with different concentrations of DMOG (activator of HIF‐1α) and 2ME2 (inhibitor of HIF‐1α) for 6 hours, and the expression level of miR‐126 was determined using RT‐qPCR. We found that DMOG significantly increased the expression level of HIF‐1α and miR‐126 in a concentration‐dependent manner in normoxia (Figure 5A‐B). Meanwhile, 2ME2 significantly decreased the expression level of HIF‐1α and miR‐126 in a concentration‐dependent manner in hypoxia (Figure 5C‐D). To further investigate the relationship of HIF‐1α and miR‐126 under hypoxia, we analysed and compared the expression of HIF‐1α and miR‐126 in HUVECs treated with DMOG and 2ME2, respectively, under hypoxia. The results showed that the expression of HIF‐1α and miR‐126 had a high degree of consistence in their changing tendencies (Figure 5E‐F). Meanwhile, the change tendency in PIK3R2 and SPRED1 protein levels were inversely proportional to miR‐126 expression under the same conditions (Figure 5G‐H). Taken together, these results confirmed that HIF‐1α could trigger expression of miR‐126 in HUVECs both in normoxia and hypoxia.

### 3.5 Effect of miR‐126 on hypoxia‐induced tube formation and angiogenic signalling pathway of HUVECs

**Figure 6:**
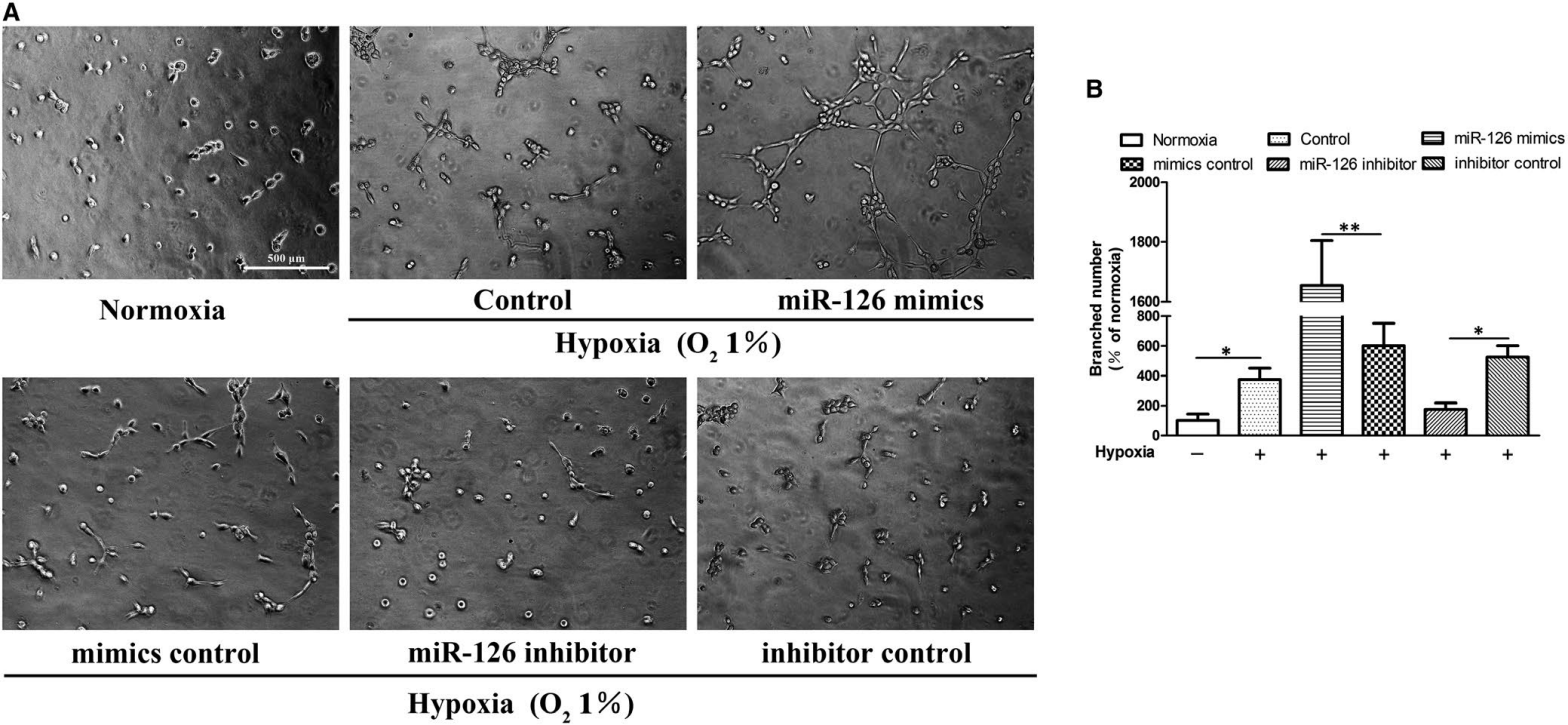
miR‐126‐mediated tube formation of HUVECs. Cells were treated 4 hours in normoxic condition (21% O_2_, 5% CO_2_) or hypoxic condition (1% O_2_, 5% CO_2_ and 94% N_2_). (A) Images of tube formation assay in all groups. (B)The branched numbers analysed by ImageJ (normoxia group was set to 100%). Values are expressed as mean ± SD (n = 3/group), *P < .05; **P < .01

**Figure 7:**
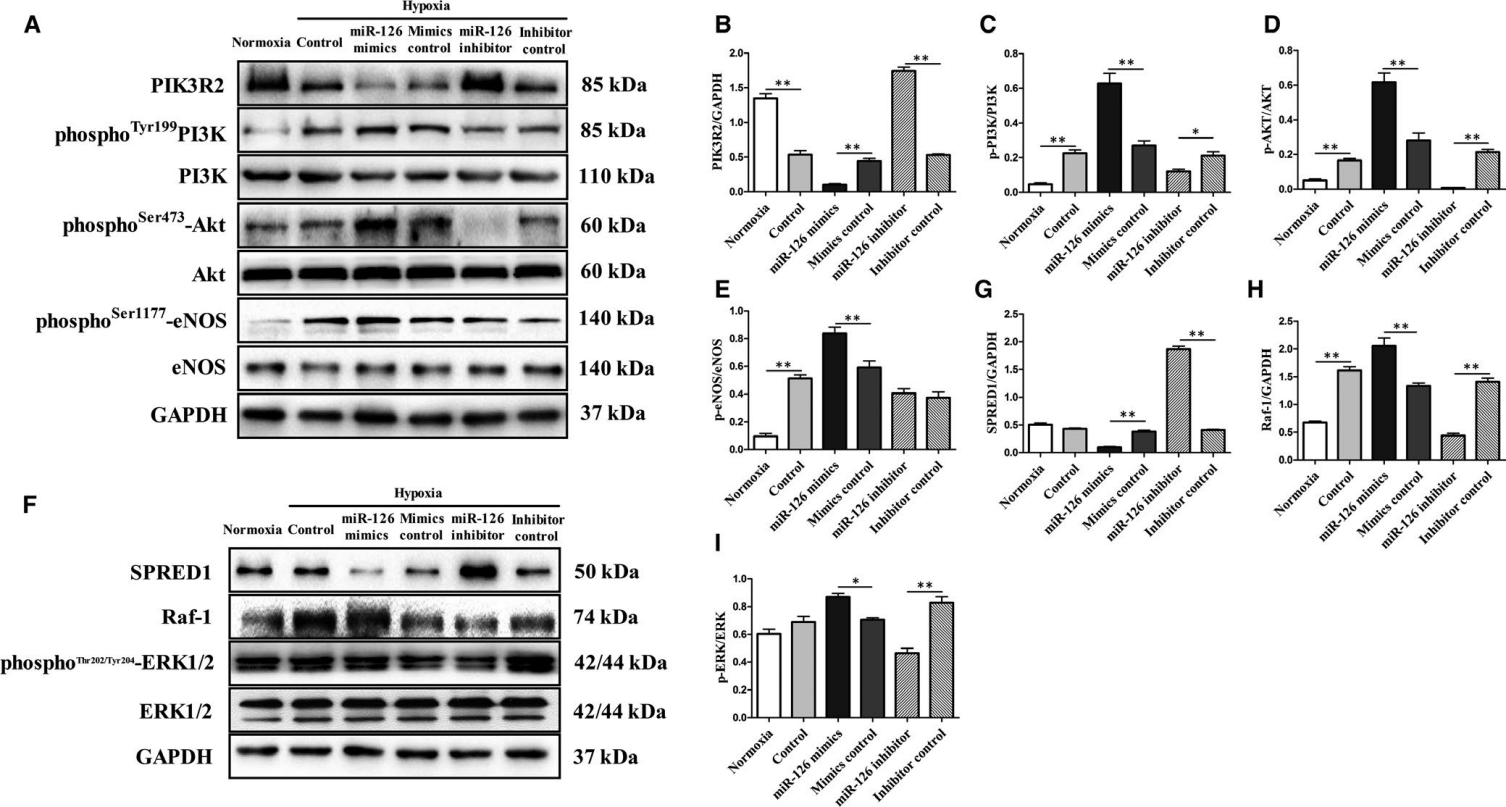
miR‐126‐mediated PI3K/AKT/eNOS and MAPK signalling pathway via PIK3R2 and SPRED1 of HUVECs. HUVECs were transfected with miR‐126 mimics, miR‐126 inhibitor and their control vehicles respectively in hypoxia for 2 hours. (A‐E) Western blot images and quantitative analysis of PIK3R2 protein content and phosphorylation levels of PI3K, AKT and eNOS (n = 3/group). (F‐I) Western blot images and quantitative analysis of SPRED1, Raf‐1 protein contents and phosphorylation levels of ERK1/2 (n = 3/group). Values are expressed as mean ± SD, *P < .05; **P < .01

To explore the role of miR‐126 in HUVECs, we overexpressed and silenced the expression of miR‐126 in HUVECs under hypoxia. Branched numbers in miR‐126 mimics group were significantly increased compared with mimics control group, while branched numbers in miR‐126 inhibitor group were significantly reduced compared with inhibitor control group (Figure 6A‐B). Cardiac PIK3R2 and SPRED1 protein expressions were significantly reduced (*P* < .01) in miR‐126 mimics group and significantly increased (*P* < .01) in the miR‐126 inhibitor group compared with the control group (Figure 7A‐B,F,G). Angiogenesis was known to be partly mediated by PI3K/AKT and MAPK signal pathways. Therefore, we measured the phosphorylation status of PI3K, AKT, eNOS and ERK to assess the PI3 kinase and MAP's activation. Quantitatively, compared with the mimics control group, p‐PI3K/PI3K, p‐AKT/AKT and p‐eNOS/eNOS were significantly higher in miR‐126 mimics group (Figure 7A,C‐E). Compared with the inhibitor control group, p‐PI3K/PI3K and p‐AKT/AKT were significantly lower in miR‐126 inhibitor group (Figure 7A,C‐D). In addition, compared with the mimics control group, Raf‐1 protein level and p‐ERK/ERK were significantly higher in the miR‐126 mimics group (Figure 7F,H‐I). Compared with the inhibitor control group, Raf‐1 protein level and p‐ERK/ERK were significantly lower in the miR‐126 inhibitor group (Figure 7F,I). Together, these data suggested that miR‐126 may be involved in the tube formation of HUVECs under hypoxia through the PI3K/AKT/eNOS and MAPK signalling pathway.

## 4 DISCUSSION

The present study revealed that HIF‐1α played an important role during ET‐induced myocardial angiogenesis and cardiac function in MI rats, thus providing new insights on the rehabilitation mechanism of ischaemic heart diseases. Furthermore, miR‐126, which can be triggered by HIF‐1α, could promote the tube formation through angiogenic signalling pathways in HUVECs, which may be the molecular mechanism underlying the influence of HIF‐1α signalling pathway on the cardioprotection.

Exercise training has received increasing attention as natural, non‐drug cardioprotective stimuli that can induce prolonged or sustainable cardioprotective states.^5^ Many factors contribute to the beneficial effects of exercise in maintaining cardiovascular system health and delaying the progress of heart diseases.^24^ The beneficial effects of exercise may be associated with anabolic/catabolic balance, calcium handling and myocardial fibrosis.^25^, ^26^, ^27^ Recently, myocardial angiogenesis has been recognized as one of the innovative therapeutic methods for the treatment of MI.^28^ Angiogenesis, in which new blood vessels form from the existing vascular network by proliferation and migration of endothelial cells, is an important rehabilitative strategy validated in multiple forms of cardiac disease animal models.^29^ Our results showed that ET could significantly increase the number of PCNA^+^/vWF^+^ cells, suggesting that ET could promote myocardial angiogenesis in MI hearts. However, HIF‐1a inhibitor, 2ME2, abolished ET‐induced myocardial angiogenesis and cardioprotection. This implies that HIF‐1α may play a core role during the ET‐induced myocardial angiogenesis in MI rats.

It has been established that the cellular response to exercise is achieved, to a large extent, by activation of HIF‐1,^30^ which functions as a master regulator of oxygen homeostasis by controlling both the delivery and utilization of O_2_. HIF‐1 is a heterodimer composed of a HIF‐1α subunit and a constitutively HIF‐1β subunits.^31^ Of the two subunits, the expression of HIF‐1α is tightly regulated by O_2_ and targets a wide array of genes.^9^, ^32^ More than 2% of all human genes are directly or indirectly regulated by HIF‐1 in arterial endothelial cells,^33^ among which vascular endothelial growth factor (VEGF) is the most representative one. By promoting the blood vessel regeneration, it can build the myocardial microcirculation of infarct area.^34^ Chen et al^35^ confirmed the existence of hypoxic reactive miRNAs in vascular endothelial cells, and some miRNAs regulated by HIF‐1 were also investigated in other cell models.^36^, ^37^ Interestingly, previous study had highlighted the critical role of miR‐126, partly functioning by directly repressing negative regulators of the VEGF pathway, including the PIK3R2 and SPRED1.^16^ Our recent research also found that ET could significantly up‐regulate the expression of myocardium miR‐126, suppress the expression of PIK3R2/SPRED1 and promote angiogenesis in peri‐infarct zone.^19^ Consequently, we hypothesized that there may be a functional interaction linking HIF‐1α and miR‐126. In the present study, DMOG and 2ME2 significantly up‐regulated and down‐regulated the expression levels of miR‐126 in HUVECs in the concentration‐dependent manner compared with the untreated group. These findings indicated that HIF‐1α could induce the expression of miR‐126 in HUVECs.

Angiogenesis is a complex process precisely regulated by multiple signalling pathways and molecules.^38^ Hundreds of genes involved in different steps of angiogenesis are independently responsive to hypoxia. miR‐126, highly expressed in vascular endothelial cells, is considered a master regulator of physiological angiogenesis.^15^ Vascularization of the injured myocardium can be found in wild‐type post‐MI mice. In contrast, there was a relative paucity of new vessels in the miR‐126 null mice.^16^ Transplantation of mesenchymal stem cells transfected with miR‐126 can improve angiogenesis and cardiac function in the infarcted area of the hearts of mice.^39^ Thus, miR‐126 appears to carry significant weight for normal neovascularization in the wake of MI. In our findings, mimics of miR‐126 performed better than the control group in the hypoxia‐induced tube formation, while the anti‐miR‐126 decreased the level of tube formation. The miR‐126 has two validated targets: PIK3R2 and SPRED1, which function as negative regulators of VEGF signalling pathways PI3K and MAPK, respectively. To confirm the role of miR‐126 in angiogenic signalling pathway under hypoxia, we studied not only the protein expression of PIK3R2 and SPRED1 after miR‐126 inhibition or miR‐126 overexpression, but also the expression changes of phosphorylated PI3K, Akts, eNOS and ERK1/2. All the results confirmed that miR‐126 was indispensable in the hypoxia‐induced tube formation of HUVECs, which could be achieved by direct regulation of its targets that worked on angiogenic pathways, such as MAPK and PI3K/Akt/eNOS.

There are several limitations in the present study. First of all, constrained by the success rate of MI model establishment, sampling sites and exercise equipment in this study, the sample size was relatively small. In this regard, more samples and multicentre studies should be employed to consolidate our current findings. Besides, we did not use miR‐126 gene knockout rats to observe the effects of exercise on myocardial angiogenesis and cardiac function, which would have enabled more direct and authentic observation of the results. Furthermore, besides myocardial angiogenesis, there are various mechanisms involved in the exercise‐induced cardioprotection of MI hearts, including myocardial inflammation,^40^, ^41^, ^42^ apoptosis^43^ and oxidative stress,^44^, ^45^ among which crosstalk may occur. Therefore, further studies are needed for investigation.

In summary, we demonstrated that HIF‐1α, whose expression experienced up‐regulation during ET, could function as an upstream regulator to miR‐126, resulting in angiogenesis promotion through the PI3K/AKT/eNOS and MAPK signalling pathway and consequent improvement of the MI heart function. This study provides evidence for what we believe is a novel molecular pathway underlying the ET‐induced cardiac repair. The importance of miR‐126 may be considered as a potential candidate for therapeutic approach against angiogenesis‐related disorders.

## CONFLICT OF INTEREST

All authors approve final version of manuscript and declare that there is no conflict of interest.

## AUTHOR CONTRIBUTIONS

**Wei Song:** Conceptualization (lead); Data curation (lead); Formal analysis (lead); Investigation (lead); Methodology (equal); Project administration (equal); Resources (equal); Software (lead); Supervision (equal); Validation (equal); Writing‐original draft (lead); Writing‐review & editing (lead). **Qiaoqin Liang:** Data curation (supporting); Investigation (supporting); Methodology (supporting); Software (supporting); Validation (equal). **Mengxin Cai:** Data curation (supporting); Formal analysis (supporting); Methodology (supporting); Writing‐review & editing (supporting). **Zhenjun Tian:** Conceptualization (equal); Funding acquisition (lead); Investigation (supporting); Methodology (equal); Project administration (lead); Resources (lead); Supervision (equal); Validation (equal); Writing‐original draft (supporting); Writing‐review & editing (equal).
